# ANXA2 in cancer: aberrant regulation of tumour cell apoptosis and its immune interactions

**DOI:** 10.1038/s41420-025-02469-x

**Published:** 2025-04-15

**Authors:** Le Huang, Kailing Xu, Qingping Yang, Zijun Ding, Zhenduo Shao, Enliang Li

**Affiliations:** 1 Department of General Surgery, The Second Affiliated Hospital, Jiangxi Medical College, Nanchang University, 1 Minde Road, Nanchang, 330006 China; 2 HuanKui Academy, Nanchang University, Nanchang, 330006 Jiangxi China; 3 Department of Reproductive Medicine, the First Affiliated Hospital, Jiangxi Medical College, Nanchang University, No. 17, Yongwai zheng Street, Nanchang, Jiangxi 330006 China; 4 School of Ophthalmology and Optometry, Nanchang University, Nanchang, 330006 Jiangxi China; 5 Jiangxi Provincial Key Laboratory of Intelligent Medical Imaging, Nanchang, Jiangxi China

**Keywords:** Tumour biomarkers, Oncogenes

## Abstract

Annexin A2 (ANXA2) is a multifunctional protein that binds to calcium and phospholipids and plays a critical role in various pathological conditions, including cancer and inflammation. Recently, there has been increasing recognition of the significant role of ANXA2 in inhibiting apoptosis and promoting immune evasion in tumour cells. Therefore, a deep understanding of the regulatory mechanisms of ANXA2 in tumour cell apoptosis and its relationship with immune evasion can provide new targets for cancer therapy. This review summarizes the role and mechanisms of ANXA2 in regulating apoptosis in tumour cells, the connection between apoptosis regulation and tumour immunity, and the potential role of ANXA2 in therapy resistance.

## Facts

- ANXA2 plays a significant role in modulating tumour cell resistance to apoptosis by interacting with pathways related to ROS, autophagy, the DNA damage response, and glucose metabolism.
- The regulatory role of ANXA2 in apoptosis is intertwined with mechanisms that modulate tumour immunity, suggesting its dual role in promoting survival and immune evasion in tumour cells.
- ANXA2 facilitates tumour cell resistance to therapies like radiotherapy and chemotherapy by regulating tumour cel apoptosis.
- Inhibiting ANXA2 may enhance apoptosis and improve tumour cell sensitivity to existing therapies, offering a promising avenue for cancer treatment development.

## Open questions

- How does ANXA2 precisely interact with apoptotic pathways, such as ROS, autophagy, and glucose metabolism, to regulate tumour cell apoptosis?
- What roles does ANXA2 play in bridging the regulation of tumour cell apoptosis and tumour immune evasion?
- How does ANXA2 induce therapeutic resistance by regulating tumour cell apoptosis?
- What are the roles of ANXA2 as a biomarker and potential target in cancer therapy?

## Introduction

ANXA2 is a calcium-dependent phospholipid-binding protein that plays important roles in various biological processes, including membrane repair, redox, and signal transduction [1]. Among them, membrane repair and the antioxidant response involve adaptive regulation of ANXA2 in response to mechanical and oxidative stress [2, 3], and this regulatory mechanism helps tumour cells resist apoptosis induced by stress signals. Apoptosis is a programmed cell death process observed in mammals that mainly eliminates aged, damaged or abnormal cells and maintains the normal function of tissues through the extrinsic pathway (death receptor-mediated) or the intrinsic pathway (mitochondria-mediated) [4, 5]. The intrinsic pathway is triggered by intracellular stress signals (such as DNA damage and oxidative stress), whereas the extrinsic pathway is triggered by extracellular signals, such as Fas ligand (FasL), tumour necrosis factor-alpha (TNF-α), and tumour necrosis factor-related apoptosis-inducing ligand (TRAIL) [6], which subsequently initiates a series of molecular events. In tumour cells, however, apoptosis-related pathways are often aberrantly regulated, leading to uncontrolled tumour growth and treatment resistance [7, 8]. For example, radiotherapy induces apoptosis by inducing DNA damage and reactive oxygen species (ROS) production; however, tumour cells acquire therapeutic resistance by inhibiting apoptosis through an enhanced DNA damage response and ROS stress [9, 10]. Therefore, sensitivity to and adaptive changes in these stress signals play important roles in tumour cell resistance to apoptosis. Although several studies have demonstrated the roles of ANXA2 in tumour progression, no systematic review has elucidated the roles of ANXA2 with respect to antiapoptotic effects, how ANXA2 responds to apoptotic stress signals, or its relationship with immune escape. Therefore, this review elucidates the roles and mechanisms of ANXA2 in regulating apoptosis in tumour cells from the perspectives of ROS, autophagy, the DNA damage response, and glucose metabolism. Additionally, we discuss the connection between this regulation of apoptosis and tumour immunity, as well as a potential role of ANXA2 in resistance to tumour cell therapy and its promise as a therapeutic target for cancer treatment.

## Mechanism of ANXA2 regulation and activation

### The structure and normal physiological functions of ANXA2

**Fig. 1:**
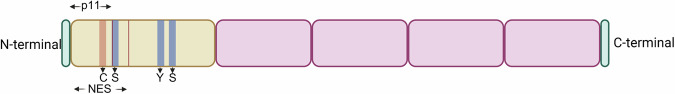
Structure of ANXA2. C represents the cysteine residue at position 8, S represents the serine residues at positions 11 and 25, and Y represents the tyrosine residue at position 23. The amino-terminal (N-terminal) contains the S100A10 (p11) binding site and a nuclear export signal (NES). The carboxy-terminal (C-terminal) includes four repeated amino acid sequences.

ANXA2, a member of the annexin family, is a slightly curved 36 kDa protein that consists of an amino-terminal variable structural domain (N-terminal) and a carboxy-terminal core structural domain (C-terminal) [11] (Fig. 1). Further studies revealed that the N-terminus of ANXA2 contains multiple binding active sites that are relatively unique features of membrane-bound proteins, including phosphorylation sites for Ser-11, Ser-25, and Tyr-23; ligand‒protein interaction sites such as the p11 (S100A10 fragment) binding site; and the nuclear export signal (NES) as well as the reactive cysteine residue Cys [12–14]. The phosphorylation site is activated by steroid receptor coactivator (Src) family serine and tyrosine kinases, thereby participating in the regulation of the subcellular localization and signalling of ANXA2 [15]. In addition, the C-terminus of ANXA2 consists of four repeating amino acid sequences forming a curved structure with convex and concave surfaces, where the convex surface contains calcium- and phospholipid-binding sites, whereas the concave surface contains binding sites for F-actin, negatively charged phospholipids, fibronectin, RNA, and heparin [12]. ANXA2 not only exists as a monomer alone but is also able to bind to the S100A10 protein through the ligand‒protein interaction site at the N-terminal end of the head to form a heterotetramer (AIIt) [16]. ANXA2 plays key roles in subcellular localization and downstream signalling (cell proliferation, apoptosis, migration, membrane repair, and immunosuppression). Its C-terminal domain binds to negatively charged phospholipids and actin, forming a network on the membrane surface, which facilitates membrane formation, trafficking, endocytosis, exocytosis, and protein recruitment [13]. The formation of AIIt enhances the affinity for phospholipids [17]. ANXA2 is also involved in the process of membrane repair, as its N-terminal structural domain interacts with dysferlin proteins to induce membrane fusion of intracellular vesicles at the site of injury [18] and accelerates vesicle delivery to the plasma membrane by forming a complex with S100 proteins (S100A10, S100A11), which promote membrane repair [19, 20]. In addition, tyrosine phosphorylation of ANXA2 promotes its binding to actin, rearrangement of microfilaments, formation of pseudopodia and reduction of cell adhesion, thereby facilitating cell migration and spreading [21, 22].

### The mechanism of ANXA2 activation

Recently, an increasing number of studies have reported fluctuations in ANXA2 expression levels during cellular stress [23, 24], which gradually led to the realization of the roles of ANXA2 in cellular stress sensing and regulation; thus, we summarize the following points. First, starvation conditions regulate ANXA2 levels. Amino acid and serum starvation are classical autophagy-inducing stimuli, but interestingly, the levels of ANXA2 and the autophagy-associated protein LC3-II are significantly elevated during starvation, and the knockdown of ANXA2 suppresses starvation-induced autophagy [25]. Therefore, we speculate that cells rely on autophagy at least partially to provide energy during starvation and that autophagy is controlled by regulating the level of ANXA2. Second, plasma membrane damage stimulates ANXA2 expression. Plasma membrane stress involves a series of protective responses initiated by the rapid activation of membrane repair systems by cells in the face of plasma membrane damage or dysfunction, including cytoskeletal reorganization and membrane fusion [26]. Tumour cells experience increased mechanical stress when they cross the dense extracellular matrix [27], which increases the frequency of membrane damage, in addition to increased oxidative stress, which further increases the risk of plasma membrane damage. Tumour cells compensate by overexpressing annexins, including ANXA2, and members of the annexin family promote membrane fusion events and wound healing by binding to negatively charged phospholipids in the plasma membrane [26]. Furthermore, in tumour cells, ANXA2 is correlated with prognosis and the hypoxic microenvironment [28], and the hypoxic tumour microenvironment upregulates the expression of a circRNA (circADAMTS6) through expression of the transcription factors activator protein-1 (AP-1) and TAR DNA binding protein 43 (TDP43); subsequently, circADAMTS6 recruits and stabilizes ANXA2 in a proteasome-dependent manner [29], which maintains stable ANXA2 expression levels.

### Phosphorylation of the ANXA2 protein and its involvement in tumour progression

Phosphorylation is a widely studied posttranslational modification that impacts protein localization and molecular interactions and can influence protein function. The function of ANXA2 is subject to a variety of posttranslational modifications, with phosphorylation being the predominant modification. The ANXA2 protein contains three main phosphorylation sites: Ser-11 [30], Ser-25 [31] and Tyr-23 [32]. Although direct evidence of ANXA2 Ser-11 phosphorylation in vivo is lacking, this modification has been extensively studied in vitro, mainly with respect to the effect of ANXA2 phosphorylation on the interaction with S100A10. It has been shown in vitro that Ser-11 of ANXA2 is phosphorylated as a protein kinase C (PKC) substrate and that this phosphorylation inhibits the formation of the heterotetramer AIIt [33]. AIIt serves as a coreceptor for fibrinogen and tissue plasminogen activator (tPA), accelerating the conversion of fibrinogen to active fibrinolytic enzymes and thereby promoting fibrinolysis. Through a feedback mechanism, the generated fibrinolytic enzymes can indirectly activate PKC, which subsequently phosphorylates ANXA2 and inhibits its translocation to the cell surface as well as further production of fibrinolytic enzymes [34]. These findings suggest that the negative feedback inhibition of fibrinolysis is likely mediated by the phosphorylation of the Ser-11 site of ANXA2, which in turn inhibits the formation of AIIt. In contrast, ANXA2 Ser-25 phosphorylation is associated mainly with the release of particulate matter. During secretion in chromophils, ANXA2 serines, located in a specific region below the cell membrane, are phosphorylated by PKC, which subsequently connects cellular granules to the cell membrane for the release of these granules [35]. The regulation of ANXA2 phosphorylation at the Tyr-23 site plays an important role in tumourigenesis and development. First, ANXA2 Tyr-23 site phosphorylation is related to the nuclear localization of ANXA2. Typically, ANXA2 is located in the cytoplasm and cell membrane, with a small amount distributed in the nucleus. Previously, p-glycoprotein (P-gp) was shown to bind ANXA2 and promote multidrug-resistant breast cancer invasion by regulating the phosphorylation of ANXA2, and further studies revealed that P-gp-mediated phosphorylation of ANXA2 is regulated by the receptor for activated C kinase 1 (RACK1) [36]. Similarly, RACK1 acts as a scaffolding protein that mediates Src kinase phosphorylation of ANXA2 Tyr-23 (pY23), which is associated with various malignant phenotypes, including invasion, metastasis, and EMT, in drug-resistant breast cancer cells [37, 38], and consequently, we speculate that ANXA2 Tyr-23 phosphorylation promotes pY23-ANXA2 nuclear translocation, which regulates the transcription of a variety of tumour-associated factors. However, it has also been shown that Tyr-23 phosphorylation of ANXA2 promotes its localization to the cell membrane [39]: why does this phenomenon occur? We hypothesize that the formation of tetramers by S100A10 and ANXA2 itself promotes the plasma membrane localization of ANXA2 and that the complex enhances the membrane-binding ability of ANXA2. In this case, ANXA2 Tyr-23 phosphorylation mainly serves a signalling function and does not promote the nuclear localization of ANXA2. In addition to its role in nuclear localization, ANXA2 Tyr-23 phosphorylation also plays an important role in exocrine secretion. First, the phosphorylation of the Tyr-23 site promotes the stable binding of ANXA2 to lipid rafts, which are specialized membrane microregions (raft-like microregions) in the inner leaflet of the cell membrane [40]. Second, phosphorylation of the Tyr-23 site impedes the interaction of ANXA2 with the clathrin complex, thereby preventing clathrin-dependent endocytosis [40]. Instead, ANXA2 is endocytosed via a caveolae-mediated nonclathrin-dependent pathway and is transported into late endosomes and exosomes. Finally, elevated intracellular Ca2+ levels promoted the secretion of exosomes containing ANXA2. Phosphorylated ANXA2 is associated with raft-like microregions in exosomes that play key roles in protein sorting and exosome release [41]. These exosomes can be transferred between cells and are involved in intercellular communication. In addition, in recent years, an increasing number of studies have shown that phosphorylation of Tyr-24, another site of ANXA2, plays an important role in the subcellular localization and function of the ANXA2 protein. On the one hand, phosphorylation of ANXA2 Tyr-24 promotes the distribution of ANXA2 in the nucleus. The receptor tyrosine kinase (RTK) family member ephrin type-A receptor 2 subsequently phosphorylates ANXA2 at the Tyr-24 site by activating YES proto-oncogene 1 (YES1) [42]. This phosphorylation event activates ANXA2 and leads to increased nuclear distribution of ANXA2 in gastric cancer (GC) cells [43]. On the other hand, phosphorylation of Tyr-24 inhibited the binding of ANXA2 to the cell membrane; specifically, phosphorylation of Tyr-24 anchored the N-terminal structural region of ANXA2 to the C-terminal core structural region, thus hindering the membrane bridging function of ANXA2 [44]. This inhibition can be restored by the binding of S100A4 or S100A10 [44]. Indeed, most of the functional regulation of ANXA2 occurs through the synergistic action of multiple phosphorylation sites. This is because ANXA2 has multiple key phosphorylation sites, and the phosphorylation of each site affects its different biological functions. The phosphorylation of these multiple sites enables ANXA2 to perform multiple functions under different cellular environments and stress conditions, thus regulating a variety of biological processes in cells [45].

## Regulation of ANXA2 in tumour cell apoptosis

### Direct regulation of apoptosis-related genes

P53 is a DNA-specific binding tumour suppressor that induces apoptosis by regulating the transcription of the downstream apoptotic genes p21, BAX, and GADD45 [46]. In normal cells, p53 is highly ubiquitinated. When p53 is exposed to external and internal stresses (hypoxia, DNA damage, and oncogene activation), its ubiquitination is inhibited, leading to an increase in the transcription of apoptosis-associated proteins, which initiates apoptosis [47]. The p53 promoter contains a conserved AP-1-like element called the p53 factor-1 (PF-1) site [48], which binds to c-Jun to form the c-Jun/p53 complex, thereby inhibiting p53 transcription [49]. In cisplatin-resistant tumour cells, ANXA2 promotes c-Jun N-terminal kinase (JNK) phosphorylation, and p-JNK induces c-Jun/p53 complex formation, thereby reducing p53 expression and inhibiting apoptosis [50]. Sirtuin 6 (SIRT6) is a nicotinamide adenine dinucleotide (NAD)-dependent deacetylase, and its acetylation directly regulates ANXA2 expression. In addition, SIRT6 protein levels are regulated by the ubiquitin-protein ligase E3A (UBE3A), which induces SIRT6 degradation by ubiquitinating the highly conserved Lys160 residue on SIRT6. UBEC3A also directly regulates the level of ubiquitination of p53, which leads to apoptosis inhibition through proteasomal degradation of p53 [51].

The nuclear factor kappa-B (NF-κB) signalling pathway is also regulated by ANXA2. The NF-κB complex consists of heterodimers, including the NF-κB1 (p50/p105)/RelA (p65) complex and the NF-κB2 (p52/p100)/RelB complex [52]. In the resting state, NF-κB inhibitory protein (IκB) interacts with NF-κB and masks the nuclear localization signal on NF-κB [53]. Intracellular ANXA2 is upregulated under hypoxic or stress conditions and competes with IκB to bind the p50 or RelA subunit of NF-κB, promoting the separation of the IκB-NF-κB complex and release of the nuclear localization signal, followed by the translocation of ANXA2 with the p50/RelA complex into the nucleus, which can promote the transcription of DNA damage-related genes and apoptosis-related genes such as Bcl-2, thereby regulating apoptosis and drug resistance [54, 55]. Another study reported that ANXA2 can also regulate the NF-κB pathway by altering p65 protein levels [29].

### Indirect regulation of apoptosis in tumour cells

#### Reduction of reactive oxygen species levels

AIIt is localized to the plasma membrane through the core structural domain of ANXA2, which is bound to membrane phospholipids [56]. This complex is involved in the regulation of a variety of cellular processes, including apoptosis. Apoptosis is genetically controlled autonomous programmed cell death that plays a key role in the clearance of excess or abnormal cells. In the case of apically extruded cells, for example, to maintain the integrity of epithelial cells at high cell densities, cells are often extruded from the tip to alleviate the crowding of the cellular arrangement [57], and apoptosis occurs in apically protruding cells as a result of physical stresses such as an increase in ROS [58]. ROS are important apoptosis-inducing agents that can be induced by promoting the phosphorylation of p38 mitogen-activated protein kinase (p38MAPK) to induce apoptosis [59]. ANXA2 is a novel redox-regulating protein that modulates intracellular ROS levels by participating in multiple redox cycling reactions [14]. However, in cancerous apical cells, AIIt accumulates on the membrane side of the cell and can inhibit p38MAPK phosphorylation by decreasing ROS levels, thereby preventing the induction of apoptosis [60]. Therefore, how does ANXA2 respond to the ROS response? We offer the following possibilities. Peroxiredoxin-2 (Prx2) participates in redox reactions through ANXA2 under oxidative stress. Like other annexins, ANXA2 has been described as a membrane scaffolding protein that forms a network on the membrane surface and provides a recruitment platform for other proteins [13]. ANXA2 brings Prx2 and signal transducer and activator of transcription 3 (STAT3) close to each other, transfers oxidizing equivalents from Prx2 to STAT3, and forms STAT3 oxidation products [61]. Subsequently, oxidized STAT3 is reduced by the thioredoxin-1 (Trx) redox system. Reduced STAT3 can be crucial for glutathione synthesis, and blocking STAT3 significantly reduces glutathione levels, thereby increasing ROS [62]. In addition, AIIt can also exert a direct antioxidant effect, but this effect may be glutathione dependent [63]. Glutathione is an important antioxidant that neutralizes ROS and protects cells from damage caused by oxidative stress [64]. The formation of mixed disulfides by AIIt and glutathione prevents the irreversible oxidation of cysteine residues to sulfenic or sulfonic acids [2], which serves as a protective mechanism for AIIt and provides AIIt with antioxidant capacity. AIIt can exert its antioxidant effects with the aid of glutathione and then regain its activity via deglutathionylation by glutoxigenin (thioltransferases, Grxs) [2].

#### ANXA2 induces intracellular autophagy in tumour cells to inhibit apoptosis

Autophagy is an important degradation mechanism that is activated during starvation to cope with nutrient deficiency by recycling biomolecules from the organism. In contrast, the membrane structures required for autophagosome formation are derived from multiple compartments, including the endoplasmic reticulum (ER), Golgi apparatus, mitochondria, and plasma membrane. Therefore, a complete understanding of the kinetic interactions between these compartments underlying the development and activation of autophagy is crucial. ANXA2, a Ca2+-dependent phospholipid-binding protein, regulates many intracellular transport events by interacting with a variety of proteins, phospholipids, and RNAs and is involved in membrane transport and fusion-related biological processes [65], suggesting that ANXA2 is an important link in the regulation of autophagy.

The plasma membrane origin of phagosomes is dependent on autophagy-related 16-like+ (Atg16L+) vesicles, and ANXA2 promotes inwards depression of the plasma membrane to form vesicles and subsequently coordinates the homotypic fusion of Atg16L+ vesicles to expand phagocytic vesicles [66], which may be mediated by the formation of a heterotetrameric complex formed by the binding of ANXA2 to the S100 protein. After transport to the recirculating endosomes, phagosomes are generated by homotypic or heterotypic fusion with Atg9+ vesicles [67]. Previous studies have shown that increased ANXA2 localization to the cell membrane during endoplasmic reticulum stress promotes the production of Atg16L+ autophagosomes, leading to increased autophagic flow [68]. Interestingly, however, a study by Baolong Pan et al. reported that the upregulation of ANXA2 expression in cisplatin-resistant osteosarcoma tumour samples and cell lines was associated with impaired autophagy [69]. Why are such opposing results observed? Our explanation is that the spatial localization of the ANXA2 protein influences its function. The spatial localization of ANXA2 can be categorized into secreted, cytoplasmic, nuclear, and membrane-bound types [70]. The cytoplasmic ANXA2 protein binds to transcription factor EB (TFEB) and blocks TFEB entry into the nucleus. TFEB is a member of the bHLH leucine zipper family of transcription factors that drive the expression of autophagy and lysosomal genes, and blocking TFEB entry into the nucleus inhibits the expression of several genes related to autophagy and lysosomal function (ATG9B, LC3, and p62) [71]. In contrast, in triple-negative breast cancer (TNBC), upregulated circEGFR binding to ANXA2 promotes ANXA2 translocation to the plasma membrane, leading to the release of TFEB from the ANXA2-TFEB complex and causing TFEB nuclear translocation [72]. Similarly, ER stress promotes the transcription of SEC23A through the activation of STAT3, and highly expressed SEC23A interacts with the ANXA2 protein to promote its plasma membrane localization and the subsequent translocation of TFEB to the nucleus [68]. TFEB resists apoptosis by regulating lysosomal function and autophagy [73]. In addition, the interaction of S100A10 with ANXA2 indirectly induces autophagy. Dimeric S100A10 binds to two ANXA2 molecules to form AIIt. This binding form can, on the one hand, increase the affinity of ANXA2 for Ca2+ and promote plasma membrane translocation of ANXA2, thus inducing autophagy. On the other hand, it can prevent S100A10 from being rapidly degraded by ubiquitin-dependent or nondependent proteasomes. S100A10 can regulate the localization of the autophagy initiation factor UNC51-like kinase-1 (ULK1) to endoplasmic reticulum-mitochondrial contact sites (also known as mitochondria-associated endoplasmic reticulum membranes, MAMs) to induce autophagy under IFN-γ stimulation [74]. In addition, ANXA2 induces autophagy by regulating the activity of autophagy-related proteins (Atg proteins). The activation of Atg proteins usually involves the inhibition of the mammalian target of rapamycin complex 1 (mTORC1), which is currently the most studied regulatory target of autophagy [75]. Under starvation induction, ANXA2 mediates the transcriptional activation of Atg7 and enhances autophagic flow, promoting drug resistance in tumour cells. Interestingly, however, there was no significant change in autophagy after inhibition of the mTORC1 component RPTOR/Raptor; in contrast, ANXA2 and autophagy levels were significantly reduced after silencing the mTORC2 component RICTOR [76]. These findings suggest that ANXA2 mediates the autophagic response under starvation-induced autophagy through the mTORC2 pathway rather than the mTORC1-dependent pathway and that such autophagy can inhibit chemically induced apoptosis. Further investigations have demonstrated that under starvation conditions, activated mTORC2 upregulates HSPA (heat shock protein family A, HSP70) expression. The resultant HSPA-ANXA2 binary complex confers proteolytic resistance to ANXA2. Subsequently, ANXA2 facilitates heat shock factor 1 (HSF1) phosphorylation and nuclear translocation, which activates ATG7 transcription and enhances its expression, ultimately leading to increased LC3B-II production [76]. Concurrently, phosphorylated HSF1 modulates the RNA-binding protein HuR, which binds to the Rictor 3’ UTR to enhance translational efficiency. This, in turn, stimulated mTORC2 activation [77], forming an autophagy-induced positive feedback loop. However, it has also been shown that ANXA2 negatively regulates autophagy by phosphorylating mTOR [78]. This suggests that the ANXA2-mTOR interaction regulates autophagy fluctuation [79]. The pro-survival role of ANXA2 manifests via autophagy-mediated apoptosis suppression. While this protective autophagy remains reversible under transient stress, prolonged stress conditions trigger autophagy inhibition through the interaction between ANXA2 and phosphorylated mTOR, thereby preventing excessive autophagy-induced apoptosis. Notably, when stress surpasses critical thresholds, this regulatory mechanism fails, culminating in autophagy-dependent apoptotic cell death [79].

**Fig. 2:**
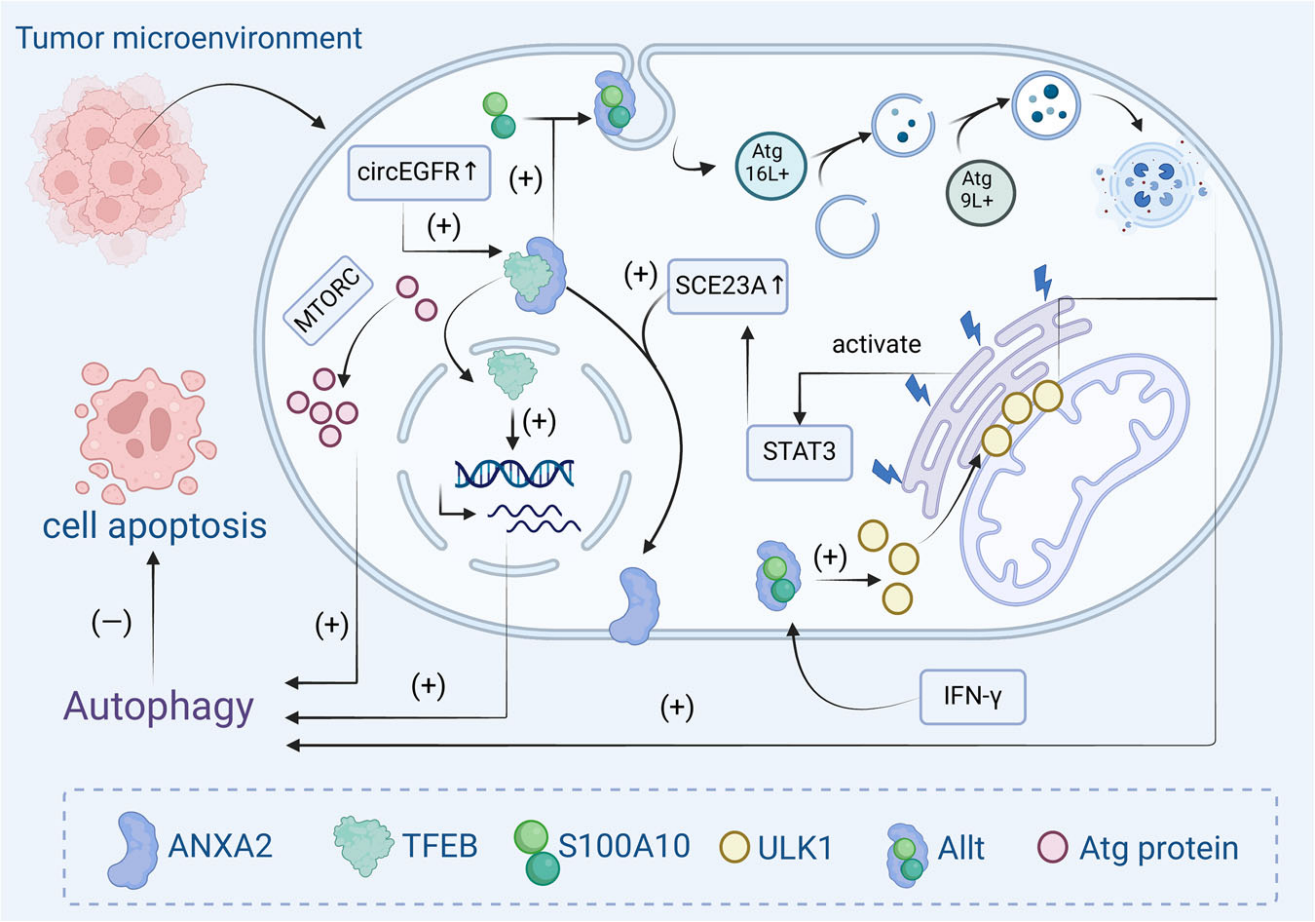
Mechanism by which ANXA2-induced autophagy inhibits apoptosis in tumour cells. ANXA2 inhibits apoptosis primarily through two mechanisms involving autophagy. On the one hand, ANXA2 enhances ATG7 transcription via an mTORC1-dependent pathway or facilitates transcription factor EB (TFEB) nuclear translocation to regulate Atg proteins, thereby modulating autophagy. On the other hand, ANXA2 induces autophagy by promoting plasma membrane invagination to form Atg16L+ vesicles or by regulating UNC51-like kinase-1 (ULK1) localization to facilitate autophagosome formation.

In summary, ANXA2 promotes autophagy and inhibits apoptosis through two main mechanisms. On the one hand, ANXA2 enhances ATG7 transcription via an mTORC2-dependent pathway or facilitates TFEB nuclear translocation to regulate Atg proteins, thereby modulating autophagy. On the other hand, ANXA2 induces autophagy by promoting plasma membrane invagination to form Atg16L+ vesicles or by regulating ULK1 localization to facilitate autophagosome formation (Fig. 2).

#### ANXA2 inhibits apoptosis by promoting glycolysis

In recent years, increasing attention has been given to the potential role of ANXA2 in metabolism, particularly glycolysis. Aerobic glycolysis is a survival strategy employed by tumour cells under normoxic conditions, wherein ATP is generated through the glycolytic pathway rather than oxidative phosphorylation, rapidly meeting the energy demands of tumour cells while increasing their stress response capabilities. The Warburg effect endows cancer cells with antiapoptotic properties. On the one hand, when the level of aerobic glycolysis in cancer cells decreases, mitochondrial oxidative phosphorylation significantly increases, leading to the production of a large amount of ROS. Increased ROS levels result in the downregulation of antiapoptotic proteins [80]. On the other hand, increased aerobic glycolysis directly affects the levels of antiapoptotic and proapoptotic proteins [81]. Therefore, promoting aerobic glycolysis is a crucial pathway for tumour cells to resist apoptosis.

The mTOR signalling pathway plays a critical role in regulating aerobic glycolysis [82]. In tumour cells overexpressing S100A10, Src-mediated phosphorylation of ANXA2 is increased, which may be related to S100A10 promoting the translocation of ANXA2 to the plasma membrane. Phosphorylated ANXA2 (p-ANXA2) subsequently phosphorylates the AKT/mTOR signalling pathway, regulating aerobic glycolysis and inhibiting apoptosis [83]. Notably, ANXA2 does not possess a kinase domain, raising the question of how ANXA2 phosphorylates Akt. In hypoxic-ischemic brain injury cells, ANXA2 phosphorylation promotes Akt phosphorylation and inhibits the mitochondria-dependent apoptotic pathway [84]. In the study by Ruiqi Liu et al., the inhibition of Akt phosphorylation by ANXA2 knockdown was reversed by the overexpression of threonine tyrosine kinase (TTK) [85], suggesting that ANXA2 likely exerts its phosphorylation effect by upregulating TTK.

Interestingly, other studies have shown that ANXA2 activation can promote an increase in mitochondrial oxidative phosphorylation levels [86]. Elevated oxidative phosphorylation implies increased entry of pyruvate into the tricarboxylic acid cycle, which relatively inhibits aerobic glycolysis, seemingly contradicting the conclusion that ANXA2 promotes aerobic glycolysis. We propose several possible explanations. First, enhanced aerobic glycolysis increases lactate production; however, the accumulation of endogenous lactate can hinder the regeneration of NAD+ [87], which in turn limits the sustained progression of aerobic glycolysis [88]. When oxidative phosphorylation is increased, NADH is oxidized to NAD+ through the electron transport chain, thereby regenerating NAD+ and maintaining glycolysis. Therefore, although aerobic glycolysis predominates in tumour cells, the role of oxidative phosphorylation cannot be ignored, as it ensures the continuous regeneration of NAD+. Additionally, ANXA2-promoted aerobic glycolysis results in the production of a large amount of lactate. In fact, lactate is not merely a metabolic waste product of aerobic glycolysis: it can directly enter mitochondria to activate the electron transport chain (ETC), thereby promoting oxidative phosphorylation, and it is also an important source of pyruvate, further participating in mitochondrial metabolism [89].

**Fig. 3:**
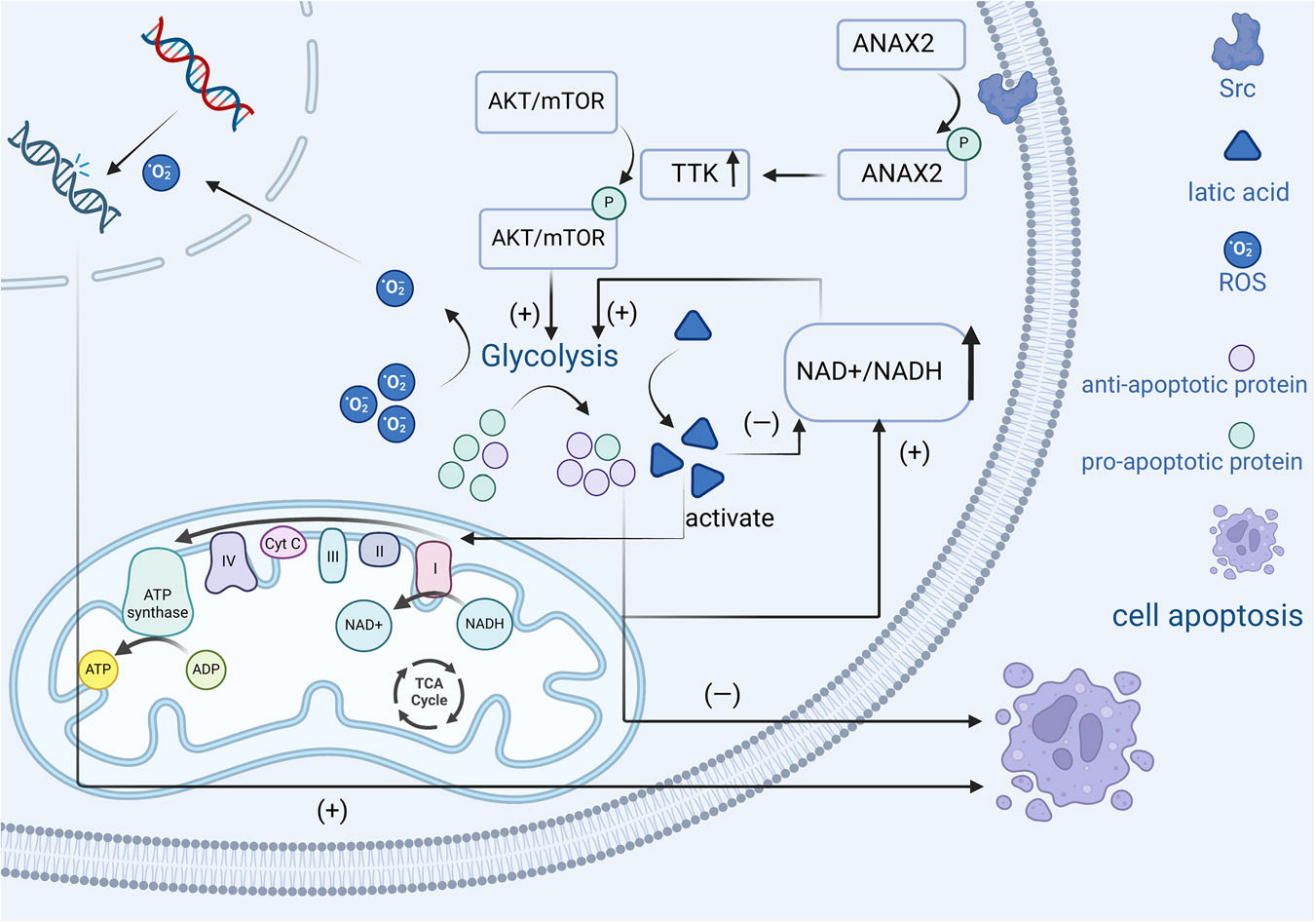
ANXA2 inhibits apoptosis by promoting aerobic glycolysis in tumour cells. Under aerobic conditions, tumour cells rapidly generate ATP through glycolysis, enhancing their anti-apoptotic characteristics. Specifically, ANXA2, upon phosphorylation by Src, promotes the phosphorylation of the AKT/mTOR signalling pathway, thereby regulating aerobic glycolysis. This action reduces the production of reactive oxygen species (ROS) and modulates the transcription of apoptosis-related genes. Moreover, the lactate produced from aerobic glycolysis can enter the mitochondria, activating the electron transport chain and promoting oxidative phosphorylation, which increases the generation of NAD+. This process helps sustain oxidative phosphorylation, further supporting the survival of tumour cells.

These studies indicate that ANXA2 inhibits ROS production and apoptosis by promoting aerobic glycolysis, but it cannot be denied that ANXA2 may also promote oxidative phosphorylation. This could be a part of the process by which ANXA2 promotes aerobic glycolysis or an effect caused by the lactate produced during aerobic glycolysis (Fig. 3).

#### ANXA2 mediates the DNA damage response and membrane repair

Under normal physiological conditions, genotoxic stress can induce nuclear accumulation of ANXA2, which contributes to the prevention of oxidative DNA damage. Compared to the control group, ANXA2 knockout cells show significantly smaller DNA repair foci after H_2_O_2_ treatment [90]. High ANXA2 expression is associated with DNA repair in a variety of cancer cells [91], and the Tyr-23 phosphorylation of ANXA2 plays an important role in cancer drug resistance [37], whereas the phosphorylation of ANXA2 Tyr-23 is closely related to nuclear localization, which implies that ANXA2 is likely a potential transcription factor for DNA repair-associated proteins. Yuning Qiu et al. demonstrated that phosphorylation of ANXA2 in the nucleus significantly activates the STAT3 signalling pathway and enhances DNA damage repair after radiotherapy and that this mechanism of radioresistance can be reversed by the STAT3 pathway inhibitor WP1066 [92], suggesting that STAT3 is an important pathway through which ANXA2 regulates DNA damage repair. Interestingly, however, ANXA2 monomers rapidly aggregated in the nucleus upon stimulation by genotoxic factors (including gamma rays, ultraviolet (UV) radiation, etoposide, and chromium VI) and H_2_O_2_. Importantly, intranuclear aggregation was mediated by inactivation of the NES and was not related to oxidation of the Cys-8 residue in ANXA2, which implies that intranuclear aggregated ANXA2 still possesses a reductively active thiol (Cys-8). Therefore, we hypothesize that ANXA2 aggregation in the nucleus not only regulates the transcription of DNA damage repair-related proteins but also directly reduces peroxides in the nucleus to prevent DNA damage. Poly ADP-ribose Polymerase (PARP) constitutes a family of NAD+-dependent enzymes that catalyze poly ADP-ribosylation (PARylation) reactions, transferring ADP-ribose units to target proteins to mediate DNA repair [93]. However, under specific pathological conditions, PARP can undergo proteolytic cleavage by effector enzymes, resulting in functional inactivation and subsequent apoptosis potentiation. Previous studies have demonstrated that ANXA2 knockdown significantly enhances PARP cleavage [50], but the precise regulatory mechanism between ANXA2 and PARP remains elusive. Based on ANXA2’s established biological functions, we propose three potential regulatory pathways. First, ANXA2 modulates cyclin D1 expression through STAT3 phosphorylation [94]. Given that cyclin D1 depletion elevates PARP cleavage and induces apoptosis [95], this suggests ANXA2 may regulate PARP via the STAT3-cyclin D1 axis. Second, oxidative stress-induced DNA strand breaks activate PARP [96]. Uncontrolled oxidative stress leads to PARP hyperactivation and subsequent cell death [97]. ANXA2’s redox regulatory capacity may maintain ROS homeostasis, thereby preventing pathological PARP overactivation. Third, ANXA2 potentially inhibits caspase-mediated PARP cleavage through modulation of apoptosis-regulatory proteins. Additionally, as a novel HSP27-interacting partner [98], ANXA2 may coordinate stress-responsive pathways. During UVC exposure, ANXA2 facilitates nucleotide excision repair (NER) pathway activation, enhancing DNA damage repair efficiency and conferring cellular resistance to UVC-induced genotoxicity [99].

Given the ability of membrane-bound protein A2 to aggregate into liposomes, we asked whether ANXA2 could promote membrane repair by aggregating at sites of damage [18]. Cancer cells are subjected to greater mechanical stress from the extracellular matrix during invasion; thus, the cell membrane of invasive tumour cells is more susceptible to damage, which leads to massive calcium ion influx and cell death. Previous studies have shown that ANXA2 and S100A11 aggregate and reseal the plasma membrane at the site of tumour cell membrane damage and that calcium ion efflux is essential in this process, which is consistent with our hypothesis. Second, the lysosomal membranes of cancer cells are more fragile than those of normal cells, which may lead to lysosomal membrane permeabilization (LMP) and the release of lethal hydrolytic proteases into the cytoplasmic lysate. Consistent with this, through a calcium-dependent membrane repair response, ANXA2 is selectively recruited to lysosomes with significant damage and mediates the resealing of lysosomal membranes upon damage to restore lysosomal membrane integrity, thereby inhibiting the initiation of apoptosis [3, 100]. When excessive intracellular ROS are produced, the opening of the mitochondrial permeability transition pore (mPTP) is induced, leading to mitochondrial swelling or even membrane rupture and the release of cytochrome C and mitochondrial DNA (mtDNA), leading to mitochondrial dysfunction and apoptosis. In addition, ANXA2 was found to promote mitochondrial membrane fusion by regulating optic atrophy 1 (OPA1) expression in non-small cell lung cancer, thereby enhancing mitochondrial metabolic adaptation and preventing apoptosis [86]. Although there is no evidence that ANXA2 can directly mediate mitochondrial membrane repair, several studies have shown that the regulation of ANXA2 phosphorylation is closely related to the membrane localization of mitochondria and that this localization affects the tumourigenicity of tumour cells [101].

## ANXA2 mediates immune regulation in the tumour microenvironment

### ANXA2-mediated regulation of immune cells

Plasmin serves as a critical signaling molecule in immune regulation. Clinical evidence indicates that plasmin contributes to immunosuppressive phenotypes, partially through cytokine modulation [102]. The AIIt heterotetramer facilitates plasmin generation through cell surface binding of plasminogen and tPA. Plasmin-mediated cleavage of ANXA2 into monomeric subunits induces dissociation of the ANXA2-S100A10 heterotetramer [103]. This proteolytic processing not only disrupts AIIt’s plasminogen receptor function but also modulates monocyte/macrophage chemokine expression via cleavage products. ANXA2 downregulation specifically impairs plasmin-dependent monocyte chemotaxis [104], establishing ANXA2 as a plasmin-induced immune cell recruitment mediator. Notably, ANXA2 knockdown suppresses macrophage M2 polarization [105], suggesting its dual roles in orchestrating macrophage trafficking to tumour sites and promoting immunosuppressive M2 differentiation. Moreover, AIIt heterotetramer is associated with inhibition of langerhans cell (LC) maturation, decreasing helper T cell 1 (Th1) cytokine secretion and reducing major histocompatibility complex class II (MHC-II) surface expression in HPV16-treated LCs exposed to AIIt. ANXA2 deficiency exacerbates proinflammatory responses, evidenced by elevated cytokine levels and enhanced neutrophil infiltration in knockout models [106]. Additionally, spatial regulation of intercellular adhesion molecule-1 (ICAM-1) through ANXA2 binding prevents neutrophil transendothelial migration. Specifically, ANXA2 deficiency prevents ICAM-1 translocation to caveolin-1-rich membrane microdomains, resulting in enhanced neutrophil adhesion and transendothelial migration [107]. This demonstrates ANXA2’s role in spatially organizing ICAM-1 distribution to control neutrophil recruitment during immunosuppressive tumour microenvironment remodeling.

Immune correlation analyses reveal ANXA2 positively associates with tumour-associated macrophages (TAMs), regulatory T-cells (Tregs), and myeloid-derived suppressor cells (MDSCs) infiltration [108], while inversely correlating with activated natural killer (NK) cells and dendritic cells (DCs) [109]. T-cell aggregation is tPA/plasmin dependent, and ANXA2 knockdown abolishes plasmin-enhanced T-cell clustering [110], highlighting the importance of ANXA2 in this process. As previously mentioned, AIIt promotes the generation of plasmin, which subsequently cleaves AIIt. The cleaved ANXA2 monomer increases F-actin formation, potentially enhancing intercellular adhesion and facilitating T-cell aggregation [110]. In NK/T-cell lymphoma (NKTCL), the GNAQ T96S mutant confers apoptosis resistance through Src kinase-dependent ANXA2 phosphorylation [111]. Additionally, human γδ T-cells, which bridge innate and adaptive immunity, recognize molecules on the surface of stressed cells via the T cell receptor (TCR), forming the first protective barrier. ANXA2, as a Vγ8Vδ3 TCR ligand, is exposed on the membrane under tumour stress, activating Vδ2neg γδ T-cells to mediate immune surveillance [24], suggesting ANXA2 as a potential immunotherapy target.

### ANXA2 as a bridge between apoptosis and immune suppression

**Fig. 4:**
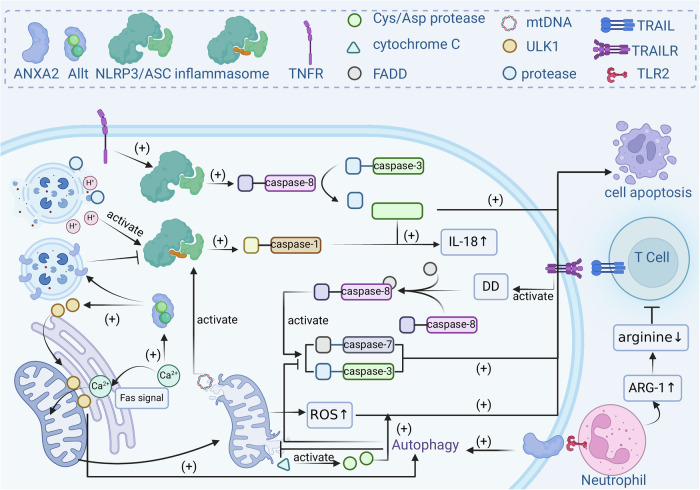
Mechanism by which ANXA2 inhibits tumour cell apoptosis and its effects on tumour immunity. Innate immunity. Lysosomal damage is a key pathway for NLRP3 inflammasome activation, with ANXA2 recruited to the damaged lysosomal membrane to facilitate repair, thereby inhibiting inflammasome activation and IL-18 release. Moreover, autophagy can interact with NLRP3 via mitochondrial-associated membranes (MAMs). Fas-mediated apoptotic signalling enhances calcium influx into the mitochondria, activating NLRP3 through mitochondrial dysfunction. The ANXA2/S100A10 complex, in response to calcium signalling, promotes the translocation of ULK1 to MAMs, inducing autophagy to clear damaged mitochondria and suppress apoptosis and inflammatory responses. Adaptive immunity. ANXA2 suppresses T-cell functions. It limits tumour cell apoptosis induced by TRAIL by promoting autophagy, which inhibits the formation of the death-inducing signalling complex (DISC) necessary for T-cell-mediated apoptosis. Additionally, ANXA2 activates TLR2 on tumour-associated neutrophils, enhancing the expression of arginase 1 (ARG1), an enzyme that depletes L-arginine in the tumour microenvironment. This depletion hampers T-cell proliferation and function, allowing tumour cells to evade immune responses.

The tumour microenvironment manifests a complex interplay between immune responses and the regulation of cell death, particularly autophagy and apoptosis [112]. On the one hand, apoptotic cells release immunogenic molecules such as damage-associated molecular patterns (DAPMs), which are recognized and taken up by antigen-presenting cells, thereby activating the immune system [113]. On the other hand, the cytotoxic effects of immune cells rely on apoptosis, including the death ligand/death receptor system [114]. Given the significant role of ANXA2 in inhibiting tumour cell apoptosis, we sought to explore whether and how this inhibition of apoptosis by ANXA2 affects tumour immunity (Fig. 4).

#### Innate immunity

The NOD-like receptor protein 3 (NLRP3) inflammasome is an intracellular multiprotein complex primarily composed of NLRP3, the adaptor apoptosis-associated speck-like protein containing a caspase recruit domain (ASC), and pro-caspase-1. It serves as a crucial innate immune sensor [115]. Upon activation, the NLRP3 inflammasome triggers pyroptosis through caspase-1 activation. Interestingly, under TNF-α stimulation, NLRP3 associates with ASC to activate caspase-8 instead of caspase-1 [116], leading to caspase-3 cleavage and apoptosis. Notably, both pathways can promote the release of IL-18 [116]. Lysosomal damage is a key pathway for NLRP3 inflammasome activation. At high concentrations of particulate wear debris, LMP occurs, causing protease and hydrogen ion leakage into the cytoplasm, which directly or indirectly activates the NLRP3 inflammasome, triggering inflammation and innate immune responses [117, 118]. However, studies have shown that ANXA2 can be recruited to damaged lysosomal membranes and facilitate their repair [100], thereby inhibiting inflammasome activation and IL-18 release. Previously, we noted that ANXA2 induces autophagy under specific conditions. Interestingly, recent studies have revealed interactions between autophagy and the NLRP3 inflammasome in cancer cells. Mitochondria-associated membranes (MAMs) are contact sites between the ER and mitochondria that are mediated by proteins and play a role in both autophagosome formation [119] and inflammasome activation [120]. Under Fas-mediated apoptotic signalling, calcium ions are transferred from the ER to mitochondria, causing mitochondrial dysfunction [121], which induces apoptosis and promotes ROS generation, activating NLRP3. Notably, calcium ions promote the formation of the ANXA2/S100A10 complex [122], and the localization of ULK1 to MAMs is regulated by the ANXA2/S100A10 complex [74]. ULK1, a serine/threonine kinase, plays a critical role in autophagy. Under calcium signalling, the ULK1 complex is activated and translocates to MAMs [123, 124]. ULK1 then regulates the activity of autophagy-related proteins, promoting autophagosome assembly and maturation [125]. Given that the C-terminal core domain of ANXA2 contains calcium-binding sites and that calcium binding enhances ANXA2’s affinity for phospholipids, we propose that calcium ions mediate autophagy at least in part by promoting the formation of the ANXA2/S100A10 complex and regulating ULK1 localization at MAMs. Notably, under IFN-γ stimulation, ANXA2 knockdown reduces ULK1 localization at MAMs and autophagosome formation, which can be reversed by S100A10 overexpression [74], indicating that ANXA2 is not necessary for ULK1 translocation to MAMs. This autophagic process helps clear damaged or dysfunctional mitochondria, reducing ROS production and inhibiting apoptosis and NLRP3 inflammasome activation. Mitochondrial apoptosis is influenced by the integrity of the inner and outer mitochondrial membranes. Upon cytochrome C release into the cytoplasm, caspases are activated, inducing apoptosis. Other factors, such as mtDNA, activate NLRP3 inflammasome signalling, promoting inflammatory responses and antitumour immunity [126].

#### Adaptive immunity

ANXA2 not only inhibits tumour cell apoptosis but also suppresses T-cell immune functions. TRAIL is a trimeric protein belonging to the TNF family that is expressed primarily on the surface of cytotoxic T cells and NK cells and acts as an extrinsic activator of apoptosis [127]. Upon T-cell activation, T-cell-derived TRAIL binds to the death receptor TRAILR on the cell surface, forming a trimer. The intracellular death domain (DD) of the receptor is then activated, recruiting Fas-associated death domain protein (FADD) and caspase-8 to form the death-inducing signalling complex (DISC) [128]. Within the DISC, caspase-8 is activated and subsequently cleaves and activates downstream caspase-3 and caspase-7, executing apoptosis [129]. During TRAIL-induced apoptosis, autophagy activation inhibits the formation and/or activation of FADD/caspase-8 [130]. Thus, ANXA2 can limit T-cell-derived TRAIL-mediated tumour cell killing by inducing autophagy, which is consistent with the findings of Silvia von Karstedt et al. [131]. Arginase 1 (ARG1) is a key enzyme that catalyses the hydrolysis of L-arginine, which is primarily released by tumour-associated myeloid cells in the tumour microenvironment. In non-small cell lung cancer (NSCLC), tumour-derived ANXA2 activates Toll-like receptor 2 (TLR2) receptors and downstream signalling on tumour-associated neutrophils (TANs), subsequently promoting ARG1 expression [132]. ARG1 depletes arginine in the tumour microenvironment, inhibiting T-cell proliferation and function. This immunosuppressive effect allows tumour cells to evade immune system attacks. Furthermore, Listeria-based ANXA2-targeted cancer immunotherapy sensitizes pancreatic ductal adenocarcinoma (PDAC) to treatment with the immune checkpoint inhibitor anti-PD-1 antibody, reshaping the immune microenvironment [133]. Therefore, ANXA2 is a promising target for cancer therapy.

## ANXA2-induced resistance through anti-apoptotic mechanisms

Radiotherapy and chemotherapy are standard adjuvant treatments for most cancer patients after surgery and promote cell death by inducing oxidative stress, destabilizing DNA, and inhibiting cell proliferation. However, our previous investigations revealed that ANXA2 plays a crucial role in resisting cancer cell death, and studies have shown that high ANXA2 expression in cancer cells is often associated with resistance to radiotoxicity and chemotoxicity. Therefore, we aimed to further explore the role of the antiapoptotic mechanisms of ANXA2 in cancer cell resistance to radiotherapy and chemotherapy.

Radiotherapy uses high-energy radiation to destabilize and break DNA structures or ionize water to produce free radicals that damage DNA, inducing cell death [134]. Chang-Yu Chen et al. demonstrated that ANXA2 nuclear translocation is a key pathway that protects cancer cell DNA from radiotherapy-induced damage [135]. The ANXA2 protein, with an N-terminal NES regulated by phosphorylation, predominantly resides in the cytoplasm and plasma membrane under normal conditions. Upon radiation stimulation, ANXA2 Tyr-23 phosphorylation is induced, inhibiting the NES signal and promoting ANXA2 nuclear translocation [92]. The exact mechanism by which nuclear ANXA2 promotes DNA damage repair remains unclear, but we propose two possible explanations. First, ANXA2 possesses redox activity. The Cys-8 residue of ANXA2 is a redox-sensitive cysteine that is reversibly oxidized by ROS (H_2_O_2_) and reduced by the thioredoxin system. Thus, ANXA2 may exert antioxidant effects by scavenging radiotherapy-induced ROS, which is consistent with studies showing that genotoxic factors promote ANXA2 monomer accumulation in the nucleus to mitigate DNA damage [90]. Second, ANXA2 knockdown significantly increases PARP cleavage [50]. Therefore, we speculate that nuclear ANXA2 may promote DNA damage repair by inhibiting PARP1 cleavage and increasing tumour cell survival.

Additionally, many targeted therapies and DNA-damaging agents can induce autophagy [136, 137]. However, in some cases, anticancer drugs induce cytoprotective rather than cytotoxic autophagy, leading to treatment resistance [138]. For example, in cisplatin-resistant human lung cancer A549R cells, ANXA2 and autophagy-related proteins are significantly upregulated, and this upregulation is associated with apoptosis inhibition [139]. Ginsenoside Rh2 (G-Rh2) treatment inhibits autophagy and increases apoptosis, but ANXA2 overexpression reverses G-Rh2 inhibition [140]. Furthermore, ANXA2 inhibits cisplatin-induced apoptosis through the activation of the JNK‒p53 signalling pathway [50]. Cancer stem cell formation is another critical factor in tumour cell treatment resistance. Under cytotoxic chemotherapy, breast cancer stem cells (BCSCs) respond to chemotherapy-induced hypoxia inducible factor-1 (HIF-1)-dependent S100A10 expression. S100A10 forms a tetramer with ANXA2, which translocates to the nucleus and interacts with suppressor of Ty 6 (SPT6) and lysine demethylase 6 A (KDM6A). This complex is recruited to the octamer-binding transcription factor 4 (OCT4) binding site, followed by KDM6A, which erases the H3K27me3 chromatin marker and promotes the transcription of genes encoding the NANOG, SOX2, and KLF4, maintains stem cell pluripotency and upregulates antiapoptotic genes, thereby inhibiting chemotherapy-induced apoptosis [141].

## ANXA2-based biomarkers and targeted cancer therapy

### Clinical significance of ANXA2 as a cancer biomarker

ANXA2, a calcium-dependent phospholipid-binding protein, exhibits dysregulated expression across multiple malignancies, with its levels strongly correlating with patient survival, prognosis, and therapeutic response. Accumulating evidence identifies ANXA2 as a pivotal biomarker for invasive breast cancer detection. In breast cancer patients, ANXA2 expression is markedly elevated in tumour tissues and serum compared to normal controls, with the most pronounced upregulation observed in TNBC subtypes [142]. Elevated ANXA2 expression correlates with advanced clinical stage (III-IV), lymph node metastasis, and positive associations with HER2 and Ki67 levels [143], implicating its role in tumour proliferation and invasion. Prognostically, elevated serum ANXA2 (especially exosomal-derived ANXA2) levels serve as an independent risk factor for reduced overall survival (OS; hazard ratio HR = 2.802) and disease-free survival (DFS; HR = 7.934) in TNBC patients, with heightened significance in African American cohorts [144]. Notably, ANXA2 secretion depends on Tyr-23 phosphorylation, and phosphorylated ANXA2 levels in TNBC tissues and serum significantly exceed those in other subtypes [142], reinforcing its diagnostic specificity. Small extracellular vesicles (sEVs) carrying ANXA2 protein/mRNA predict chemotherapy response, where high sEV-ANXA2 levels in neoadjuvant responders correlate with advanced TNBC ( ≥ Stage III) [145]. These findings collectively position ANXA2 as both a novel biomarker and potential therapeutic target. The expression and clinical significance of ANXA2 in various cancer are summarized in Table 1.

**Table 1:** The expression and clinical significance of ANXA2 in various cancers.

Cancer types	Sample	Expression	Clinical correlation	References
Breast cancer	Tissue, serum	Up	High stage, lymph node metastasis, poor prognosis	[143]
Ovarian cancer	Plasma	Up	Diagnostic value	[161]
Endometrial cancer	Tumor tissues	Up	High recurrences, metastasis	[162]
Cervical cancer	Public database	Up	Poor prognosis, invasion and migration	[163]
Lung cancer	Public database	Up	Stemness, EMT, metastasis signature, and poor clinical outcomes	[164]
Gastric cancer	Tumor tissues	Up	High clinical stage, lymph node metastasis	[165]
Pancreatic cancer	Tumor tissues	Up	Poor prognosis	[166]
Hepatocellular carcinoma	Public database	Up	Poor prognosis	[167]
Colorectal cancer	Tumor tissues	Up	Advanced stage, metastasis	[168]
Glioma	Public database	Up	Poor survival status	[108]
Oral squamous cell carcinoma	Tumor tissues	Up	TNM stage, lymph node metastasis, poor survival	[169]
Renal cell carcinoma	Tumor tissues	Up	poor survival	[170]
Bladder cancer	Public database	Up	High grade, poor OS, PFS and DSS	[171]
Poorly differentiated prostate cancer	Tumor tissues	Up	High grade and stage, earlier biochemical recurrence	[172]
Prostate cancer	Tumor tissues	Down	Less recurrence, metastasis and better prognosis	[173]
Acute lymphoblastic leukemia	Bone marrow, blood	Up	Phosphorylation and glucocorticoid resistance	[174]

### ANXA2 as a potential cancer therapeutic target

Targeting ANXA2 in cancer therapy not only inhibits tumour cell growth but also enhances the effects of chemotherapy and immunotherapy, presenting a broad application potential. The core mechanisms include down-regulation of ANXA2 protein or mRNA levels, blocking the interaction of ANXA2 with other proteins, inhibition of ANXA2 activation, and immune-targeted therapy.

Studies reveal that suppressing ANXA2 expression or its mRNA significantly inhibits tumour cell growth and metastasis. For instance, miR-101 binds the 3’-UTR of ANXA2 mRNA to suppress translation, reversing P-glycoprotein-mediated multidrug resistance and enhancing cisplatin-induced apoptosis [146]. Furthermore, ANXA2 interacts with diverse signaling molecules to drive tumour progression. Ginsenosides Rg5 and Rk1 block ANXA2-NF-κB p50 interaction, inhibiting NF-κB pathway activation and tumour cell proliferation [147]. Additionally, ANXA2 forms a heterotetrameric complex with S100A10. The first small-molecule ANXA2 inhibitor, 5α-epoxypropiolactone (5α-EAL), disrupts ANXA2-S100A10 function, impairing breast cancer stem cell self-renewal [148]. Post-translational regulation of ANXA2 presents novel therapeutic opportunities. All-trans retinoic acid (ATRA) inhibits peptidylprolyl cis/trans isomerase, NIMA-interacting 1 (PIN1)-mediated phosphorylation of ANXA2 at Tyr-23, thereby reducing cholangiocarcinoma metastasis [149]. In glioblastoma, NASP promotes the nuclear translocation of phosphorylated ANXA2, which activates STAT3 and contributes to radiotherapy resistance. This phenotype can be reversed by the STAT3 inhibitor WP1066 [92].

Advances in ANXA2-targeted therapies leverage its tumour-specific surface expression. The anti-ANXA2 monoclonal antibody mAb150 reactivates cancer stem cell cycling by recognizing the N-terminal epitope, disrupting tumour dormancy [150]. In drug delivery, ANXA2-specific aptamers exhibit unique advantages. The ACE4 aptamer, selected via Cell-SELEX, binds ANXA2 and inhibits its function across cancer models, particularly in MCF-7 cells [151]. ANXA2-targeted DNA/RNA hybrid nanoparticles conjugated with a thioaptamer enable precise doxorubicin delivery to ovarian tumours, improving therapeutic efficacy and reducing off-target toxicity [152].

Despite compelling preclinical evidence of the therapeutic potential of ANXA2-targeting strategies in inhibiting tumour growth, overcoming drug resistance, and improving efficacy, these approaches are still largely in the experimental research phase. Critical translational questions remain, including insufficient pharmacokinetic characterisation of ANXA2 inhibitor candidates and the unproven safety of systemic therapeutic targeting of this multifunctional protein. Therefore, more investigations are needed to support their clinical therapeutic efficacy and to advance further clinical studies.

## Unrevealed problems

This review provides a detailed elucidation of the mechanisms by which ANXA2 inhibits tumour cell apoptosis. We investigated how ANXA2 functions to suppress apoptosis in tumour cells and subsequently promotes tumour cell growth and treatment resistance by counteracting the apoptosis induced by radiation and chemotherapy. Additionally, we explored the relationships between tumour cell apoptosis mechanisms and cellular inflammation and immunity and how ANXA2 influences tumour cell inflammation and immunity through its antiapoptotic mechanisms. Building on these mechanistic insights, we further discuss the clinical potential of ANXA2 in prognosis and cancer therapy.

However, many unresolved questions remain regarding the role of ANXA2 in regulating tumour cell apoptosis and immune mechanisms. In this review, we clarify ANXA2 as a regulator of apoptosis, highlighting one mechanism by which ANXA2 promotes autophagy in tumour cells to inhibit apoptosis. Interestingly, some studies have shown that elevated levels of ANXA2 and autophagy are associated with increased apoptosis [153], indicating that ANXA2-induced autophagy is not always cytoprotective. We hypothesize that whether ANXA2-induced autophagy in cancer cells is beneficial for resisting apoptosis depends on the extent of autophagy. When tumour cells experience stress responses such as nutrient stress induced by the microenvironment, ANXA2 activation and upregulation induce autophagy, promoting the degradation of intracellular components to provide energy and cope with starvation, which is reversible and protective autophagy. However, prolonged stimulation or excessive autophagy under starvation conditions can damage essential organelles, triggering irreversible and cytotoxic autophagy [79]. Therefore, it can be inferred that tumour cells inhibit apoptosis through moderate autophagy activation by ANXA2.

In the review, we describe how ANXA2 regulates apoptosis in tumour cells through its effects on ROS, autophagy, DNA damage response, and glycolysis. However, these pathways appear relatively independent, thus, we propose two possible synergistic regulatory axes that unify their functions. First, ANXA2 establishes a dynamic equilibrium linking ROS homeostasis, aerobic glycolysis, and DNA repair via its redox regulatory and protein recruitment capacities. Its reducing activity directly scavenges intracellular ROS through redox-sensitive cysteine residues, mitigating mitochondrial dysfunction [154, 155], while STAT3 recruitment drives Cyclin D1 phosphorylation to activate hexokinase 2 (HK2)-mediated aerobic glycolysis [156]. Enhanced glycolytic flux suppresses mitochondrial electron leakage, limiting ROS generation and fueling tumour proliferation while bolstering antioxidant defenses. Upregulated Cyclin D1 additionally suppresses PARP cleavage-induced apoptosis [95], whereas ANXA2-mediated ROS containment prevents ROS-dependent apoptosis and PARP-overactivation cascades under oxidative stress [96, 97]. Second, ANXA2 orchestrates ROS-autophagy-metabolic crosstalk to establish multilayered resistance. ROS-induced ANXA2 upregulation enhances lysosomal biogenesis and autophagic flux, eliminating damaged mitochondria (mitophagy) and oxidized proteins to curb ROS accumulation [157]. This autophagy-dependent ROS clearance synergizes with ANXA2’s direct antioxidant function, while mitophagy-driven oxidative phosphorylation reduction shifts energy production toward glycolysis [158]. By promoting the synergistic action of glycolysis and autophagy, ANXA2 ensures a balance between energy supply and cellular clearance efficiency.

Furthermore, the roles of ANXA2 in tumour inflammation and immunity remain poorly understood. IL-1, particularly IL-1β, plays a crucial role in initiating and maintaining inflammatory responses within the tumour microenvironment. This chronic inflammation in tumours promotes the accumulation of T-cell-suppressive neutrophils and other immunosuppressive cells through the continuous release of protumour inflammatory factors, which is consistent with the conclusion that ANXA2 mediates IL-1β production and promotes tumour immune suppression [159]. Our previous findings indicated that ANXA2 inhibits NLRP3 inflammasome activation through lysosomal repair and autophagy induction, thereby suppressing pyroptosis. Notably, NLRP3 inflammasome activation plays a central role in IL-1 production and release. How does ANXA2 promote IL-1 production and secretion? Typically, IL-1β secretion requires inflammasome activation to cleave pro-IL-1β into mature IL-1β, followed by the formation of membrane pores by gasdermin D (GSDMD) to facilitate the release of IL-1β. Interestingly, studies have shown that the release of bioactive IL-1β within tumours does not depend on the activation of inflammasomes, caspase-8, the pore-forming protein GSDMD, or mixed lineage kinase domain-like protein (MLKL) [160]. These findings suggest that inflammasome activation is not essential for IL-1β secretion. Therefore, we speculate that ANXA2 may promote IL-1β maturation and release through noninflammasome-dependent pathways. One possible mechanism is that ANXA2 activates the NF-κB signalling pathway, increasing the expression levels of other proteases that cleave pro-IL-1β and promote its maturation. However, no studies have directly revealed how ANXA2 promotes IL-1β maturation and release. Further research is needed to explore this mechanism in depth.

Despite the many mysteries surrounding the role of ANXA2 in regulating tumour cell apoptosis and immune mechanisms, we hope to provide new perspectives for understanding the complex functions of ANXA2 in tumours from the perspective of apoptosis. We aim for these findings to offer new insights into targeted cancer therapy strategies and inspire more in-depth research on ANXA2 in tumour biology.
